# Interventions to address the mental health of adolescents and young adults living with or affected by HIV: state of the evidence

**DOI:** 10.1002/jia2.25713

**Published:** 2021-06-24

**Authors:** Arvin Bhana, Philip Kreniske, Ariana Pather, Melanie Amna Abas, Claude A Mellins

**Affiliations:** ^1^ Health Systems Research Unit South African Medical Research Council Durban South Africa; ^2^ Centre for Rural Health College of Health Sciences University of KwaZulu‐Natal South Africa; ^3^ HIV Center for Clinical and Behavioral Studies Department of Psychiatry New York State Psychiatric Institute and Columbia University New York NY USA; ^4^ Institute of Psychiatry, Psychology and Neuroscience King’s College London London UK

**Keywords:** HIV, mental health, adolescents and young adults, psychosocial interventions

## Abstract

**Introduction:**

Adolescents and young adults (AYA) remain vulnerable to HIV‐infection and significant co‐morbid mental health challenges that are barriers to treatment and prevention efforts. Globally millions of AYA are living with HIV (AYALH) and/or have been affected by HIV in their families (AYAAH), with studies highlighting the need for mental health programmes. With no current guidelines for delivering mental health interventions for AYALH or AYAAH, a scoping review was undertaken to explore current evidence‐based mental health interventions for AYALH and AYAAH to inform future work.

**Methods:**

The review, targeting work between 2014 and 2020, initially included studies of evidence‐based mental health interventions for AYALH and AYAAH, ages 10 to 24 years, that used traditional mental health treatments. Given the few studies identified, we expanded our search to include psychosocial interventions that had mental health study outcomes.

**Results and discussion:**

We identified 13 studies, seven focused on AYALH, five on AYAAH, and one on both. Most studies took place in sub‐Saharan Africa. Depression was targeted in eight studies with the remainder focused on a range of emotional and behavioural symptoms. Few studies used evidence‐based approaches such as Cognitive Behaviour Therapy; psychosocial approaches included mental health treatments, group‐based and family strengthening interventions, economic empowerment combined with family strengthening, group‐based mindfulness and community interventions. Eleven studies were randomized control trials with four pilot studies. There was variation in sample size, treatment delivery mode (individual focus, group‐based, family focus), and measures of effectiveness across studies. Most used trained lay counsellors as facilitators, with few using trained mental health professionals. Eleven studies reported positive intervention effects on mental health.

**Conclusions:**

Despite the need for mental health interventions for AYALH and AYAAH, we know surprisingly little about mental health treatment for this vulnerable population. There are some promising approaches, but more work is needed to identify evidence‐based approaches and corresponding mechanisms of change. Given limited resources, integrating mental health treatment into healthcare settings and using digital health approaches may support more standardized and scalable treatments. Greater emphasis on implementation science frameworks is needed to create sustainable mental health treatment for AYALH and AYAAH globally.

## 1 Introduction

In 2019, 1.7 million people were newly infected with HIV, with 460,000 of these new infections in adolescents and young adults (AYA), ages 10 to 24‐years [1, 2]. The number of new AYA infections is largely driven by horizontal transmission and 9% of new infections still occur in children 14 years and older, primarily through vertical transmission [2]. Moreover, improvements in antiretroviral therapy (ART) have resulted in children with perinatally acquired HIV (PHIV) surviving into adolescence and adulthood. AYA with PHIV experience unique challenges to those with behaviourally acquired HIV, including developmental delays, neurocognitive issues, and not finding out their diagnosis until older. Furthermore, sub‐populations of AYA experience unique vulnerabilities, for example, adolescent girls and young women accounted for 25% of new infections in sub‐Saharan Africa in 2019, despite making up about 10% of the total population [1]. In the United States (USA), young men who have sex with men, followed by young transgender women are at greatest risk for HIV‐infection, particularly Black and Latinx populations. These same groups are also at high risk in many Asian and Pacific countries [3]. While many countries are reporting excellent progress towards HIV prevention, recent data confirm a bleak picture globally for AYA. Older adolescents and younger adults (15 to 24 years) compared to children and young adolescents (0 to 14 years) and older adults (25+ years) have the lowest chance of knowing their status and the lowest likelihood of HIV viral suppression – along with the highest HIV incidence [4]. These data are being hidden in health information systems which pool figures in all those over the age of 14, and in those which merge older and younger adolescents [5]. Given the high burden of HIV among AYA, addressing the differential emotional and behavioural needs and complex vulnerabilities of AYA living with HIV (AYALH) is critical to treatment and prevention.

Mental health challenges are a critical barrier to ending the HIV epidemic across contexts and populations, given that they increase the risk of non‐adherence to ART, poor engagement in care and sexual and substance use risk behaviours for HIV transmission [6, 7, 8, 9]. Studies from multiple countries increasingly show high rates of various mental health problems in AYALH ranging from psychosocial distress and symptoms of emotional and behavioural problems to actual psychiatric disorders [7, 9, 10, 11, 12, 13, 14], with some studies finding higher rates of mental health problems in youth compared to those with PHIV [15, 16].

Importantly, addressing mental health problems early in AYA development is crucial for the wellbeing of those impacted [17] given that more than 50% of adult mental health disorders appear before the age of 14 years globally [18, 19, 20]. During this period, AYA experience extensive physical, hormonal, neurocognitive and psychosocial transformations. These changes are challenging to manage in the context of living with a chronic, life‐threatening, stigmatized and sexually transmissible virus, that can have significant neurological effects [7, 21, 22].

Unfortunately, understanding the role of HIV and other pathways of influence on mental health has been difficult to determine. Studies of AYALH have often utilized comparison groups of AYA affected by HIV (AYAAH) ‐ including those who lost one or both parents to AIDS, or who are living in households with caregivers or family members living with HIV, and those who were perinatally HIV exposed, but uninfected (PHEU) as they share many of the same psychosocial and contextual vulnerabilities. Results show that AYAAH are also at increased risk for mental health problems [23, 24], with some studies suggesting that they are at even greater risk than AYALH [11, 14, 16]. For example, in one study, AYAAH were more likely to be depressed, anxious and report internalized stigma, suicidal ideation and excessive substance use than AYALH [16]. Importantly, both groups have shown higher rates of mental health problems than the general population [8, 25, 26]. In low‐resource contexts, where the majority of AYALH and AYAAH reside, limited availability of psychosocial supports adds to the challenges of coping with poor mental health [7, 27, 28, 29].

There are numerous individual, familial, social and environmental risks that can profoundly influence life trajectories and mental health in both AYALH and AYAAH [7, 27]. Both groups typically face ongoing and cumulative psychological stressors exacerbated by adverse environments including poverty, violence, discrimination, familial and environmental substance abuse or mental illness, as well as HIV‐related illness and loss of caregivers. Both groups also confront stigma associated with familial HIV. All these factors have been associated with behavioural problems and psychiatric disorders, including post‐traumatic stress disorder, depression and severe anxiety [30], which in turn may increase sexual risk behaviours, putting AYAAH at risk of HIV infection, and putting AYALH at risk for non‐adherence to treatment, poor health outcomes, and secondary transmission to others. For these reasons, it is imperative to consider mental health interventions for both groups.

Fortunately, over the past 20 years, substantial advances in evidence‐based mental health treatment for AYA in the general population could be a basis for intervention work with AYALH and AYAAH. Evidence‐based mental health treatment, that is a treatment approach supported by a body of research evidence relating to its efficacy, include Cognitive‐Behavioural Therapy (CBT) [31, 32], Interpersonal Psychotherapy (IPT) [33, 34], Dialectical Behavioural Therapy (DBT) and Acceptance and Commitment Therapy [35]. Most of these treatments have been evaluated with AYA in multiple trials in multiple contexts, although primarily in resource‐rich countries, led by professional therapists. Although limited, increasingly trials are being conducted for use by lay counsellors in low‐ and middle‐income countries (LMIC) [36].

Relatedly, multiple reviews have documented that even fewer trials have tested interventions which target the mental health needs AYALH or AYAAH, particularly in LMIC, with mixed findings [9, 24, 29, 37, 38]. Due to the lack of sufficient evidence, the World Health Organization guidelines on mental health treatment and prevention for adolescents (Helping Adolescents Thrive) concluded that despite the priority of delivering mental health interventions to AYALH, no recommendations could be made and highlighted a need for high‐quality research on psychosocial interventions promoting AYALH mental health [39]. A similar need has been advocated for AYAAH including uninfected AYA with PHEU given the high rates of mental health problems and potential risks of perinatal exposure to ART, as well as similar psychosocial and contextual risks as AYALH [40]. Given the research gap, a scoping review was undertaken to explore current evidence‐based mental health interventions for AYALH and AYAAH (including uninfected AYA with PHEU) and to explore how research gaps could be addressed to inform evidence‐based interventions for this population.

## 2 Methods

We followed the Preferred Reporting Items for Systematic Review and Meta‐Analysis (PRISMA) guidelines for scoping reviews [41], searching data in August and September 2020 using online electronic databases, including PubMed (which includes Medline), the following EBSCO databases Psycharticles, Socindex, Academic Search Complete, Family and Society Studies Worldwide; CINAHL; Web of Knowledge Social Science and Emerging sources databases; EMBASE; Proquest; Scopus; Cochrane Reviews; Epistemonikos (evidence‐based healthcare database); CT.gov (http://apps.who.int/trialsearch) and the International Clinical Trials Registry Platform (https://clinicaltrials.gov). In addition, we examined the National Institute for Health databases and the AIDS 2020 Virtual Conference Oral Abstracts and E‐posters conference proceedings. Lastly, additional hand searching, and citation tracking was done.

Given previous reviews [6, 7, 8, 9, 24, 29, 37, 38, 42], we focused our search on 2014 to 2020. We predefined our search using Population, Intervention, Comparison, Outcome and Study (PICOS).

- Population: AYALH or AYAAH, aged 10 to 24 years. Although adolescence is typically defined as ages 10 to 19 years, there is inconsistency in the definition across agencies and contexts. Additionally, in many clinical HIV settings, AYA remain in paediatric HIV care into their twenties (19 to 24 years). While it is recognized that several stages of development exist within adolescence itself (early – 10 to 13 years; middle – 14 to 16 years; late – 17 to 19 years), many studies do not distinguish between these stages, and thus we included studies using a broader definition of adolescence. This broad range ensured inclusion of a larger number of studies, with the recognition that younger and older adolescents may require different approaches.
- Intervention: any mental health treatment approach, or psychosocial intervention that has promotion of mental health or reduction of mental health distress as an outcome. This could include a range of modalities, including school‐based, social and family support, community and economic interventions, etc.
- Comparison group: any choice of comparison group including no comparison group.
- Outcome: any mental health outcome variable including psychiatric disorders, general mental illness or specific mental health problems (e.g. depression, anxiety); also included were the following: psychological or emotional wellbeing, psychosocial capital or resilience, or mental or emotional health; psychological distress or emotional distress or trauma.
- Study design: any research study design that included a mental health‐related intervention. Clinical notes or reports or case‐based descriptions of mental health interventions were excluded.
- Geographic location: all global locations were included. Even though a majority of AYALH and AYAAH live in sub‐Saharan Africa, given the global epidemiology of HIV in women which in turn affects youth, we included evidence‐based mental health treatments from across the globe.

The first author (AB) undertook the scoping review of databases described above using the above criteria and the search terms described in Appendix S1. Following the search, any duplicates were removed. The remaining titles were imported into Rayyan software to scan titles and abstracts for selection [43]. A second author (PK) independently searched 25% of the same list of titles and abstracts. This review process did not find discrepancies or additional titles for inclusion. Importantly, in the first review search, only four studies were identified using more traditional evidence‐based mental health interventions (e.g. CBT, IPT). Thus, we expanded the review to include mental health promotion interventions or interventions that targeted mental health outcomes, including psychosocial, school‐based, family strengthening and economic empowerment interventions [24, 29, 38].

For presentation, we organized the studies into several categories of interventions: individual mental health treatment; group and family strengthening; family based economic strengthening; group‐based mindfulness training; and a community‐based intervention. An analysis of the review results focuses on providing a detailed description of the mental health interventions, describing the targeted mental health conditions, the specific outcome measures used, the type of evidence‐based intervention that was used, the setting in which this intervention was applied, the training and level of expertise required to deliver the intervention, and the outcomes of the mental health intervention.

## 3 Results and Discussion

**Figure 1:**
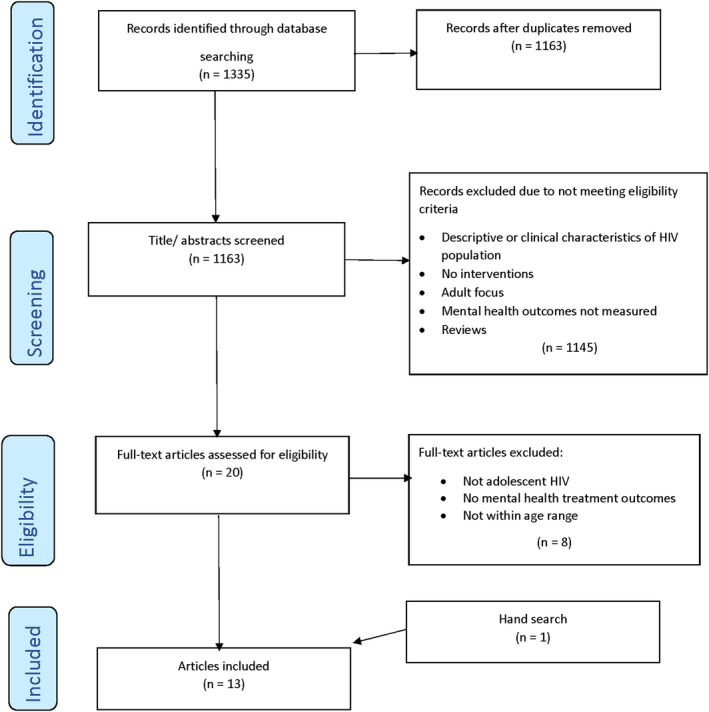
(Scoping Review) PRISMA Guide.

Our database search identified 1335 records. After removing duplicates, the remaining 1145 titles were screened. The majority did not meet study criteria as most were descriptive or clinical accounts of individuals living with or affected by HIV and focused on social, demographic and clinical characteristics rather than interventions. Only 20 titles remained for full‐text evaluation with 13 studies meeting inclusion criteria. The remainder did not have a mental health outcome, did not measure the effects of a mental health intervention, or focused on adult participants (Figure 1).

### 3.1 Evaluation of risk of bias

An evaluation of the bias risk of the 13 studies in this review was done to establish the relative strength of evidence of mental health interventions for AYALH and AYAAH using the Method for Evaluating Research Guideline Evidence (MERGE) [44]. MERGE provides a simple and well‐defined way of evaluating public health interventions. Each of the 13 articles reviewed were independently coded by two authors (AB, AP) based on criteria relevant to evaluating intervention study characteristics. An agreement of 85% was achieved with differences in scoring (two cases) resolved through discussion. Table 1 shows the overall rating for each study using MERGE guidelines. Of the 13 studies, six were very unlikely or unlikely to change in outcome, with seven likely or very likely to have changed outcomes as a function of design flaws (Table 1).

**Table 1:** Risk of bias analysis

Author and year	Comments	Overall assessment of bias	Rating	Outcomes
Betancourt *et al* (2017)	Contamination of community samples	Low‐moderate risk	B1	Unlikely to change
Cavazos‐Rehg *et al* (2020)	Well‐controlled study	Low risk of bias	A	Very unlikely to change
Dow *et al* (2020)	Some bias due to lack of blinding and differences in treatment approach	Low‐moderate risk	B1	Unlikely to change
Kennard *et al* (2014)	Case control study; small sample, no control group	High risk of bias	C	Very likely to change
Li *et al* (2014)	Small sample and potential bias in randomization process	Moderate‐High risk	B2	Likely to change
Mon *et al* (2016)	Randomized only some groups; measurement bias	Moderate‐high risk	B2	Likely to change
Nestadt *et al* (2019)	Small sample size, well controlled study	Low‐moderate risk	B1	Unlikely to change
Puffer *et al* (2016)	Potential contamination and temporal effects of stepped‐wedge design	Low‐moderate risk	B1	Unlikely to change
Ssewamala *et al* (2016)	Well controlled study	Low‐moderate risk	B1	Unlikely to change
Thurman *et al* (2018)	Biased sampling, no randomization and no control group	High risk of bias	C	Very likely to change
Vreeman *et al* (2019)	Only sites randomized, not individual allocations	Moderate‐high risk	B2	Likely to change
Webb *et al* (2018)	Small sample size and 25% loss to follow‐up	Moderate‐high risk	B2	Likely to change
Willis *et al* (2019)	Small sample size and 32% loss to follow‐up	Moderate‐high risk	B2	Likely to change

### 3.2 General characteristics

Among the 13 studies, eight focused on AYALH [45, 46, 47, 48, 49, 50, 51, 52], five on AYAAH [53, 54, 55, 56, 57] and one on both [58] (See Table 2). Nine studies took place in sub‐Saharan Africa, three in Asia (China, Myanmar, Thailand), and two in North America (both USA).

**Table 2:** Study characteristics

Author, year and country	Study population		Intervention focus and facilitators	Study design & inclusion criteria	Mental health outcomes only	Summary of mental health findings only
	HIV status‐and other criteria	Total N; gender and age				
Betancourt *et al* (2017) Rwanda No MH requirement	AYAAH and AYALH living with one HIV positive caregiver	82 families N = 170 AYAAH (7 to 17 years, mean= 11) (48% female) (12% HIV positive; n = 21) N = 123 caregivers	FSI‐HIV (Family Strengthening Intervention) to promote mental health and improve parent–child relationships in families with caregivers living with HIV Manualized, delivered by: Bachelor level counsellors; 90‐minute weekly home visiting; 8 sessions	Pilot RCT	Depression Conduct problems Functional impairment	At 3 months, AYAAH in FSI‐HIV showed fewer symptoms of depression compared to controls by self and parent report No significant differences by group on conduct problems, functional impairment
Cavazos‐Rehg *et al* (2020) Uganda No MH requirement	AYALH	N = 702 (10 to 16 years, mean=12.42) (56.4% female)	Family level economic strengthening intervention includes 4 financial management sessions, support for ART, microenterprises for family income over 24 months + 12 educational sessions and adherence counselling Delivered by lay counsellors in ART adherence + finance mentor	Repeated measures RCT	Hopelessness Depression Self‐Concept Hopelessness Depression Self‐Concept	At 24 months, treatment arm reported lower hopelessness score and at 36 months, lower depression scores
Dow *et al* (2020) Tanzania No MH requirement	AYALH	N = 128 AYALH (12 to 24 years, mean= 18) (51% female) (84% perinatally acquired)	Two individual and ten group‐based intervention sessions with elements of CBT, IPT, MI, Trauma counselling lasting 90 minutes Delivered by young adults (23 to 30 years) living with HIV and/or prior experience with mental health research	RCT – stepped wedge design	Depression Emotional and behavioural problems Stigma Post‐traumatic stress	At 6 months, no significant differences in mental health scores for depression, emotional and behavioural problems and stigma compared to the control arm
Kennard *et al* (2014) USA MH requirement	AYALH diagnosed with depression	N = 8 (16 to 24 years, mean= 21) (13% female) (88% behaviourally acquired)	14 planned, 60‐minute sessions over 6 months using health and wellness CBT intervention with elements of MI; psychotropic medication; and psychoeducation Delivered by masters/ doctoral level therapists trained in CBT	Treatment group at two treatment sites – with no control group	Depression	At 6 months, depression improved
Li *et al* (2014) China No MH requirement	AYAAH	N = 79 (6 to 12 years, 13 to 18 years (47% female)	A multilevel family intervention focusing on the family’s capacity overcome impact of living with HIV small group session, home‐based family activities and community events to promote social integration	Cluster RCT	Self‐esteem Problem behaviour Depression	At 6 months, improved self‐esteem among 6 to 12‐year‐old children, but not 13 to 18 years; decrease in problem behaviour in controls
Mon *et al* (2016) Myanmar No MH requirement	AYAAH	N = 160 AYAAH (10 to 16 years) (58.8% female)	Mindfulness training group intervention sessions over 3 months for a total of 8 sessions lasting 120 minutes each Delivered by trained mindfulness leader	Cluster RCT	Emotional and Behavioural Problems	At 6 months, emotional and behavioural problems improved; no effect on social behaviour
Nestadt *et al* (2019) Thailand No MH requirement	AYALH; on ART	N = 88 dyads of caregiver and AYALH AYALH (9 to 14 years, mean= 12) (49% female) (100% perinatally infected)	11 weekly sessions using structured manualized intervention, including a cartoon‐based curriculum, follow‐up activities and facilitated discussion over 6 months Delivered by a social worker or counsellor	Pilot RCT	Depression Emotional and Behavioural Problems	At 6 months, statistically significant improvements in emotional and behavioural problems, sustained at 9 months, but not on depression
Puffer *et al* (2016) Kenya No MH requirement	AYAAH	124 families N = 237 AYAAH (10 to 16 years, mean= 12) (51% female) N = 203 caregivers	Church‐based intervention for families of 9 sessions of 120 minutes each delivered in churches Delivered by 4 lay facilitators who received 5‐day training and weekly preparation (no prior training in MH or HIV prevention)	Cluster RCT – stepped wedge design	Self‐esteem Depression Anxiety Emotional and Behavioural Problems	At 3 months, no effects on mental health outcomes
Ssewamala *et al* (2016) Uganda No MH requirement	AYAAH; (Orphans)	N = 346 (12 to 16 years, mean= 13) (65% female)	Family level economic strengthening that included one mentorship meeting per month, managing a savings account and ten, 1 to 2 hours microenterprise skills over 12 months Delivered by lay counsellors and finance mentor	Two‐arm cluster RCT	Hopelessness Self‐concept	At 24 months, the treatment arm reported lower‐levels of hopelessness, and high‐levels of self‐concept compared to the control arm
Thurman *et al* (2018) South Africa No MH requirement	AYAAH	N = 105 AYAAH (12 to 17 years) (60% female) N = 95 caregivers	A structured curriculum building core HIV knowledge and behavioural skills (Let’s Talk) comprising 19 caregiver and 14 adolescent sessions over 90 minutes. Includes CBT elements Delivered by trained facilitators who received three one‐week training sessions	Pilot study (Pre‐post no control group)	Depression Anxiety	At 3 months, both caregivers and adolescents demonstrated improved mental health including depression and anxiety
Vreeman *et al* (2019) Kenya No MH requirement	AYALH	N = 285 dyads of caregiver and AYALH (10 to 14 years, mean = 12) (52% female)	Disclosure intervention comprises access to intensive group and one‐on‐one counselling; pamphlets and videos over 2 years Delivered by trained counsellors	Cluster RCT	Depression Emotional and Behavioural Problems	At 24 months, no significant differences between groups on depression or emotional and behavioural problems
Webb *et al* (2018) USA No MH requirement	AYALH	N = 72 AYALH (14 to 22 years, mean = 18.7) (45% female)	Mindfulness stress reduction programme (MBSR) of nine sessions, including CBT elements (present‐focus, rumination and future concerns) as well health education covering nutrition, exercise and puberty Delivered by instructors trained in MBSR over 8 weekly sessions and extensive experience teaching mindfulness and by health education instructors trained over 3 hours on curriculum and had health education experience.	Pilot RCT	Mindful Attention and Awareness Perceived Stress Coping Aggression Emotional regulation	At 3 months, significant improvement in mindfulness: positive mental health (mindfulness; problem solving coping; life satisfaction; and aggression) No significant differences in perceived stress, rumination, distraction, anxiety or cognitive flexibility
Willis *et al* (2019) Zimbabwe No MH requirement	AYALH; on ART	N = 94 AYALH (10 to 15 years) (59% female)	Improving linkage to services through Community Adolescent Treatment Supporters trained and mentored in adherence and psychosocial support; Weekly home visits Delivered by youth aged 18 to 24 trained and mentored in adherence and psychosocial support counselling	RCT	Self‐confidence Self‐esteem Self‐worth	At 12 months, significant improvements in psychosocial wellbeing (confidence, self‐esteem, and self‐worth); quality of life

CBT, cognitive behaviour therapy; FSI, family strengthening intervention; IPT, interpersonal psychotherapy; MH, Mental health; MI, motivational interviewing.

Depression was the most common mental health problem studied (N = 9) [45, 46, 47, 48, 51, 54, 56, 57, 58], with various depression scales used, including the Patient Health Questionnaire (PHQ‐9) [45, 48], the Center for Epidemiologic Studies Depression Scale for Children [58], the Child Depression Inventory [47, 51, 54], the Zung Self‐Rating Scale [57] and the Depression Anxiety Stress Scale [56]. Only one study used a formal depression diagnostic tool, the Quick Inventory of Depressive Symptomatology‐Clinician, since depression was an inclusion criterion for study participation [46].

Five studies utilized a general measure of emotional and behavioural symptoms, the Strengths and Difficulties Questionnaire (SDQ) [45, 47, 48, 53, 54], two studies assessed hopelessness [51, 55], one focused on emotional regulation [49], and one study focused on self‐esteem, self‐worth, stigma (not defined) and quality of life as indicators of mental health [50]. Apart from two studies [46, 49], the measures in all others were adapted/translated to suit local needs in terms of language and meaning. None of the studies undertook any formal validation of the measures used.

Importantly, the 13 studies reflect a range of intervention strategies, including CBT, IPT, motivational interviewing (MI), family strengthening, economic empowerment, mindfulness, problem‐solving and disclosure as presented below. Although mental health had to be an outcome for our review, it was not necessarily the only outcome – adherence, stigma and disclosure, for example were other outcomes in the studies. Moreover, the interventions were implemented by a range of facilitators from trained masters and doctoral‐level therapists with enhanced CBT skills to community adolescent treatment supporters, trained lay health workers and skilled mindfulness instructors. All the interventions included some form of facilitator training and supervision.

### 3.3 Individual mental health treatment interventions

As noted, only one study used a mental health diagnosis for study inclusion criteria [46]. This study examined the influence of depression treatment on adherence and health outcomes among eight AYALH in the USA diagnosed with depression. The intervention used CBT that included problem solving, mood monitoring, behavioural activation and cognitive restructuring to decrease obstacles to adherence and increase wellness (self‐acceptance, positive relations with others, autonomy, environmental mastery, purpose in life and personal growth). Training in emotional regulation, social skills, assertiveness and relaxation was also provided. This study found that at 24 weeks post‐baseline, the criteria for remission were met with a depression mean score of 1.5 compared to a score of 16 at baseline. Adherence improved from 74.3% to 90.5%. Although promising, with only eight participants, no comparison group and multiple components, a larger trial is needed to evaluate intervention efficacy.

Another study, based in Kenya, examined a counselling intervention to enhance HIV disclosure among AYALH randomized to intervention or control. This disclosure intervention comprised intensive counselling with trained counsellors and resulted in increased disclosure over 24 months, but with no statistically significant group differences in depression and SDQ scores [48].

### 3.4 Group and family strengthening interventions

One study used an randomized control trial (RCT)randomized control trial (RCT) and stepped‐wedge design to evaluate a combined individual (2 sessions) and group‐based (10 sessions) mental health intervention for AYALH [45]. The intervention, delivered by trained young adult group leaders who were either living with HIV and/or had prior mental health research experience, addressed mental health challenges through teaching strategies to improve coping and family support, and reduce internal stigma. The intervention was adapted for AYALH, in part, from a CBT intervention for bereaved orphans in Tanzania and included components of IPT and MI. Depression and SDQ scores improved overall, but with no statistically significant group differences. Scores on internalized stigma (effect size 2.11), externalized stigma (effect size 1.5) and self‐reported adherence scores (effect size 7.29) all improved significantly in the intervention arm compared to standard of care.

A group‐based intervention for AYAAH in Kenya was evaluated through a cluster RCT with families attending various churches [54]. The intervention sought to improve overall family communication particularly in relation to economic, emotional and HIV‐related topics. While the intervention resulted in significant improvements in family communication compared to the comparison group, there was no significant impact on AYAAH mental health.

A few studies, all in LMIC, used family strengthening interventions. One pilot RCT with 88 families in Rwanda found that AYAAH and AYALH had significantly fewer symptoms of depression after receiving a family home‐based intervention to support mental health and promote parent–child relationships compared to controls [58]. Another study used an adapted version of the Collaborative HIV and Adolescent Mental Health Program (CHAMP), an evidence‐based intervention originally developed for youth at risk for HIV in the USA and validated in a large‐scale clinical trial [59], and then adapted for AYAAH and AYALH in multiple contexts including the USA, South Africa, and Thailand (also called CHAMPSA, CHAMP+, CHAMP+Asia and VUKA) [52]. CHAMP is a developmentally timed intervention which aims to prevent HIV infection and promote mental health in adolescents through promoting family and peer support, adolescent and caregiver skill building, and promotion of resilience [59, 60, 61, 62].

Several RCT pilot studies with early adolescents living with HIV, adapted this multiple family group‐based intervention, CHAMP+ [47, 61, 62, 63]. The most recent work, CHAMP+Asia took place in several HIV clinics in Thailand and focused on the promotion of mental health and ART adherence and prevention of sexual and drug‐use risk behaviours in AYALH and can be delivered by lay counsellors, masters level psychologists or social workers. Six‐month post baseline, AYALH in CHAMP+ condition had significantly improved adherence and mental health scores on the SDQ, but not on the Child Depression Inventory, compared to the comparison group [63].

Another randomized pilot study in China evaluated a family strengthening programme, Together for Empowerment Activities, a multilevel intervention that focuses on a family’s capacity to overcome the impacts of living with HIV. It incorporates group and home‐based family activities as well as community events to promote parent–child communication and improve AYAAH self‐esteem and reduce any problem behaviour [57]. Group differences were noted in self‐esteem for children 6 to 12 years of age at three months, but not at six months, and not for adolescents [13, 14, 15, 16, 17, 18]. Both age groups showed significant group differences in the level of parental care at six months. The groups did not differ significantly on reduction in problem behaviours. A second family centred programme to mitigate HIV risk (knowledge, self‐efficacy) and promote mental health among AYAAH in South Africa, focused on building core HIV knowledge and behavioural skills in a one group pretest–posttest design [56]. Elements of the intervention incorporated CBT components (goal‐setting, challenging negative thoughts, problem‐solving skills) for adolescents and caregivers. Significant improvements were noted concerning AYAAH mental health outcomes (depression and anxiety symptoms) on the Depression Anxiety Stress Scale as well as other outcomes (e.g. HIV transmission, condom knowledge).

### 3.5 Family based economic strengthening interventions

Another approach to family based interventions involves economic strengthening. SUUBI (Hope) has been delivered in Ugandan school settings and was originally designed for AYAAH. Evaluated through a large‐scale cluster RCT [55], the SUUBI intervention group had significantly lower levels of hopelessness and improved self‐concept compared to the control group who received usual care. SUUBI, delivered by lay counsellors, has been found to be effective in reducing sexual risk behaviour and promoting emotional wellbeing in multiple countries in sub‐Saharan Africa [64, 65]. SUUBI+Adherence was adapted for AYALH, and evaluated in a RCT in multiple clinics in Uganda, with both the intervention and comparison groups also receiving bolstered psychosocial support, using a cartoon‐based curriculum on disclosure, adherence and coping similar to that used in CHAMPSA and VUKA in South Africa [60, 66, 67]. The SUUBI+Adherence trial found significant intervention effects on hopelessness and depression, at 24‐month post intervention [51].

### 3.6 Group‐based mindfulness interventions

Group‐based mindfulness interventions were tested in two studies, one in Myanmar and one in the USA. Mindfulness is increasingly being used in many contexts as part of mental health treatments, for example DBT [35] and on its own or as part of CBT [31, 32]. Derived from the Buddhist way of meditation, mindfulness is described in one of the studies as a state of consciousness in which there is enhanced attention of moment‐to‐moment experience.” (Pp 2) [53]. In the Myanmar study, structured mindfulness training delivered by an experienced mindfulness instructor and two investigators included elements of family strengthening (involving parents/ guardians in the process and to monitor homework as well as meditation. In a cluster RCT, significant improvements in three domains of psychological behaviours (emotional [effect size 1.8], conduct and social behaviour [effect size 0.81]) at six months were noted among AYAAH who received the intervention component relative to the comparison group. In the USA study, a clinic‐based pilot RCT was delivered by instructors with extensive experience in teaching mindfulness. They provided mindfulness‐based stress reduction intervention over at least eight weekly sessions with didactics on and experiential practice of various mindfulness techniques (meditation, yoga) and application of mindfulness to everyday life. Significant improvement in the intervention group in mindfulness, problem‐solving coping styles, life satisfaction, reducing aggression and lower HIV viral load was noted among African American AYALH [49].

### 3.7 Community‐based intervention

The last RCT, community‐government partnerships were leveraged to enhance linkage and retention in care for AYALH. This programme recruited young adults from the local community to be peer counsellors. Known as Community Adolescent (18 to 24 years) Treatment Supporters, they were trained and mentored by the Zimbabwean Health and Child Care Ministry [50]. The study found significant increases in psychological wellbeing (self‐confidence, self‐esteem, self‐worth), lower stigma and quality of life care, as well as improved adherence to ART and linkage to care, but not retention in care compared to controls.

In this review, in the past six years, 13 intervention studies were identified, with most conducted in sub‐Saharan Africa where the majority of AYALH and AYAAH reside. The majority of these studies (n = 10) demonstrated positive intervention effects on some component of mental health [46, 58], with only two studies of AYALH [45, 48] and 1 of AYAAH [54] showing no effect of the intervention on mental health outcomes. However, the limited number of studies with full scale trials of mental health (four studies were pilot studies), the diversity of interventions and measures used, the diversity of age groups, study periods and contexts, the different types of facilitators, and the extreme limit in evidence‐based treatments for specific mental health problems, make it very difficult to discern best practices. The risk of bias analysis indicated that seven of the 13 studies [46, 49, 50, 53, 56, 57] were likely to have outcomes that would probably change because of the bias introduced by small sample sizes or flawed randomization procedures. Furthermore, while all the interventions used trained facilitators, there was great variation in training and previous facilitator experience, ranging from highly skilled CBT therapists to lay counsellors with varying levels of supervision and training.

In three of the four studies that used evidence‐based mental health treatment [45, 49, 53], participants did not need to meet criteria for a mental disorder diagnosis, making it difficult to assess i mpact on those most vulnerable to psychiatric illness. Only one study included a formal diagnosis of participant mental health before undertaking the intervention [46]. Furthermore, studies used varying post‐intervention periods, from immediately post‐intervention to 24‐month post‐intervention, making comparisons difficult. The most commonly used measure of mental health was the SDQ which is not a diagnostic tool but a symptom checklist of emotional and behavioural difficulties and strengths. The most common specific mental health problem was depression, but different measures were used across studies.

The SUUBI economic strengthening intervention was one of the most effective approaches in promoting mental health in AYALH [51, 55]. This finding supports the increasing awareness that poverty is one of the key social determinants of poor mental health and that economic strengthening approaches can be helpful [68]. Additionally, studies focused on family strengthening interventions showed promise in addressing mental health outcomes among adolescents affected by HIV in their families [53, 54, 55, 56, 57]. The studies of mindfulness are also encouraging; mindfulness is increasingly recognized as an evidence‐informed intervention for mental health as part of other treatments such as DBT and CBT or on its own, although testing and adaptation are needed in African countries at the centre of the HIV epidemic. For interventions to have a chance of being scalable, they need to be culturally appropriate and deliverable by non‐mental health specialists, given the dearth of mental health providers in many contexts. We did not find any studies examining the capacity of lay counsellors to provide mental health treatment to AYALH or AYAAH with mental health disorders.

In Zimbabwe, a study currently underway is testing problem‐solving therapy, an evidence‐based transdiagnostic intervention for common mental disorders in youth, in a setting of high HIV prevalence, modelled on the Friendship Bench [69]. More studies are needed of transdiagnostic or simple evidence‐based interventions such as problem‐solving therapy or behavioural activation, that can be delivered by health workers, in order to promote feasibility in LMIC where capacity for psychiatric diagnosis may be limited [70].

Implementing mental health interventions in LMIC, particularly using evidence‐based mental health treatment, is challenging given the limits on funding and available professionally trained staff to conduct screening, diagnostic evaluation and the treatment itself. For example in South Africa, there is only one psychiatrist per an estimated 357,143 people [71]. As a result, many studies reviewed here utilized lay counsellors and interventions that did not require specialized professional experience and training. It has been suggested that digital technology‐based interventions could be helpful in resource‐constrained contexts in standardizing treatment and reducing training costs as well as the need for professional providers [72, 73]. A recent review suggested that digital psychological interventions (mostly with depression and substance use) are superior to control conditions and moderately effective in LMIC [74]. Mental health interventions that leverage digital technology may also provide increased access for AYA who heavily use social media and other text‐based and internet‐based platforms and be well suited for rapid scale‐up with ongoing studies offering insights for the future development and scale‐up of technology‐based mental health interventions tailored to AYALH [75, 76]. That said, many AYA in resource‐constrained contexts in particular do not own mobile phones or share phones with others, and those who do own phones may have limited access to airtime, the internet and reliable electricity [77, 78, 79, 80], requiring different approaches to treatment. Regardless of how interventions are delivered, studies to determine lay counsellor capacity, training and supervision required, and the potential role of digital technology in supporting them are needed.

Almost none of the 13 projects included descriptors of possible mediators promoting change, and most studies used multicomponent interventions. This leaves us unable to clearly identify mechanisms of change or make definitive statements about what worked and why. An analytic framework that may be useful in unpacking interventions that seek to influence complex systems is the use of realist reviews. By making explicit the underlying assumptions (programme theories) about how an intervention is meant to work with what impact, realist reviews may help in providing an explanatory analysis as to what works for whom, in what circumstances, in what way and how [81].

The risk of bias identified in many of the studies reviewed provides an opportunity for future work to ensure adequate sample size and randomization considerations in study recruitment and treatment in addressing the mental health needs of AYALH and AYAAH. It is likely that there is not a one‐size‐fits‐all approach given that mental health has multiple biological, psychological and social causes, and context is critical to what is acceptable, accessible and feasible. Implementation science research approaches may help improve our understanding of what works for whom and under what conditions [82] and can also promote scale up and sustainability of mental health interventions beyond the life span of research projects. In low‐resource contexts, the design of delivery systems using implementation science principles may close the mental health implementation gap [83]. A renewed focus on the barriers and enablers of the implementation of an intervention is called for, as well as the health or mental health impact of the intervention itself [84]. Moreover, use of community‐based participatory research methods that involve community stakeholders at every step of the intervention research process can help ensure not only that interventions address the most critical problems for communities, but also that stakeholders are involved in sustainability of the programmes themselves.

Importantly, mental health problems are one of the leading causes of the loss of disability adjusted life years globally [85]. While health agencies are calling for global investment in mental health [86] and a mental health treatment gap has been identified particularly in LMIC [87], the current review suggests this mental health treatment gap may be even wider for young people living with or affected by HIV. Furthermore, this gap among youth persists in low‐, middle‐ and high‐income contexts. Integrating screening of mental disorders that can be used by non‐specialists is recommended as one way of improving access to mental healthcare [6]. This strategy could be impactful for the mental health and wellbeing of AYALH, particularly those who experience challenges with linkage and retention to HIV care.

Given the stigma of HIV, those affected are likely not to seek care outside of HIV clinics. Hence, policies that promote the integration of mental healthcare into HIV care settings may be most effective for serving the needs of AYALH in need of mental health treatment [7, 8, 88], while also ensuring the quality of mental healthcare [89]. One promising example promoting the integration of mental health within a chronic care platform (including HIV) is the scale up of the Mental Health Integration Programme (MhINT) using an implementation science framework to identify what works for whom and under what conditions [90]. Another is the work of the Hub Accelerating Achievement for Africa’s Adolescents (Accelerate) to promote high‐quality evidence generated in a series of randomized trials to improve the lives of adolescents across multiple Sustainable Development Goals [91]. But importantly, resources, policies and research to provide a sufficient evidence base are urgently needed to address the emotional wellbeing of adolescents and young adults living with and affected by HIV globally.

This study has some limitations. First, as a scoping review, we may have missed mental health interventions in our search of the literature. Given that much of the work described in this scoping review tends to reflect work done in LMIC because of the HIV burden in these regions, this unintended bias should be noted. Second, we used a broad definition of adolescence (10 to 24 years), as many studies assess adolescences in this age range. However, we recognize that several stages of development exist within this range and note that future studies should explore the differences between these stages and design developmentally tailored mental health interventions. Lastly, there are mental health interventions for adult populations affected by HIV, as well as populations of adolescents and young adults not living with or affected by HIV or living with other chronic conditions that could be useful in the context of AYALH and AYAAH, that did not form part of this review process.

## 4 Conclusions

Given the heavy burden of disease associated with HIV, the staggering numbers of adolescents living with or affected by HIV and the high rates of mental health problems previously identified in this population, there is still a substantive need for evidence‐based mental health treatment for AYALH and AYAAH. There is a need for simple brief transdiagnostic evidence‐based interventions, which are likely to be feasible in low‐resource settings that utilize lay counsellors and that are possibly supported through digital technology. Measurement tools for mental health research in AYA need to be locally validated in the contexts in which they are applied. Future research must develop implementation theory and look at implementation outcomes such as feasibility, acceptability and fidelity, as well as effectiveness, to ensure that interventions found to be effective become part of integrated mental health services for adolescents and young adults living with and affected by HIV globally.

## Competing interests

The authors have no competing interests to declare.

## Authors’ contributions

AB, PK, AP, MA and CAM were involved in conceptualizing and drafting the manuscript. All authors have read and approved the final manuscript.

## References

[1] WHO . Guidelines on mental health promotive and preventive interventions for adolescents. Geneva: WHO; 2020. 33301276

[2] UNAIDS . UNAIDS Data 2020. Geneva: UNAIDS; 2020 [cited 2020 Dec 10]. Available from: https://www.unaids.org/sites/default/files/media_asset/2020_aids‐data‐book_en.pdf

[3] UNICEF . HIV and AIDS in aolescents. 2020. Available from: https://data.unicef.org/topic/adolescents/hiv‐aids/

[4] CDC . HIV and transgender people. 2019. Available from: https://www.cdc.gov/hiv/group/gender/transgender/index.html

[5] UNAIDS . UNAIDS Data 2017. UNAIDS; 2017.

[6] Slogrove AL , Mahy M , Armstrong A , Davies M‐A . Living and dying to be counted: What we know about the epidemiology of the global adolescent HIV epidemic. J Int AIDS Soc. 2017;20:21520. 28530036 10.7448/IAS.20.4.21520 PMC5719718

[7] Chibanda D , Benjamin L , Weiss HA , Abas M . Mental, neurological, and substance use disorders in people living with HIV/AIDS in low‐ and middle‐income countries. J Acquir Immune Defic Syndr. 1999;2014 67 Suppl 1:S54–67. 10.1097/QAI.0000000000000258 25117961

[8] Mellins CA , Malee KM . Understanding the mental health of youth living with perinatal HIV infection: lessons learned and current challenges. J Int AIDS Soc. 2013;16:18593. 23782478 10.7448/IAS.16.1.18593 PMC3687078

[9] Remien RH , Stirratt MJ , Nguyen N , Robbins RN , Pala AN , Mellins CA . Mental health and HIV/AIDS: the need for an integrated response. AIDS (London, England). 2019;33(9):1411–20. 10.1097/QAD.0000000000002227 PMC6635049 30950883

[10] Vreeman RC , McCoy BM , Lee S . Mental health challenges among adolescents living with HIV. J Int AIDS Soc. 2017;20 Suppl 3:21497. 28530045 10.7448/IAS.20.4.21497 PMC5577712

[11] Mellins CA , Brackis‐Cott E , Leu CS , Elkington KS , Dolezal C , Wiznia A , et al. Rates and types of psychiatric disorders in perinatally human immunodeficiency virus‐infected youth and seroreverters. J Child Psychol Psychiatry. 2009;50(9):1131–8. 19298479 10.1111/j.1469-7610.2009.02069.x PMC2775808

[12] Abrams EJ , Mellins CA , Bucek A , Dolezal C , Raymond J , Wiznia A , et al. Behavioral health and adult milestones in young adults with perinatal HIV infection or exposure. Pediatrics. 2018;142:e20180938. 30097528 10.1542/peds.2018-0938 PMC6317560

[13] Lee B , Chhabra M , Oberdorfer P . Depression among vertically HIV‐infected adolescents in Northern Thailand. J Int Assoc Phys AIDS Care. 2011;10(2):89–96. 10.1177/1545109710397892 21368007

[14] Smith R , Huo Y , Tassiopoulos K , Rutstein R , Kapetanovic S , Mellins C , et al. Mental health diagnoses, symptoms, and service utilization in us youth with perinatal HIV infection or HIV exposure. AIDS Patient Care STDs. 2019;33(1):1–13. 30601062 10.1089/apc.2018.0096 PMC6338457

[15] Malee KM , Tassiopoulos K , Huo Y , Siberry G , Williams PL , Hazra R , et al. Mental health functioning among children and adolescents with perinatal HIV infection and perinatal HIV exposure. AIDS Care. 2011;23(12):1533–44. 21702707 10.1080/09540121.2011.575120 PMC3387978

[16] Lynn C , Bradley‐Klug K , Chenneville T , Walsh A , Dedrick R , Rodriguez C . Mental health screening in integrated care settings: Identifying rates of depression, anxiety, and posttraumatic stress among youth with HIV. J HIV/AIDS Soc Serv. 2018;17:1–7.

[17] Sherr L , Cluver LD , Toska E , He E . Differing psychological vulnerabilities among behaviourally and perinatally HIV infected adolescents in South Africa–implications for targeted health service provision. AIDS Care. 2018;30:92–101. 29848010 10.1080/09540121.2018.1476664

[18] Patton GC , Sawyer SM , Santelli JS , Ross DA , Afifi R , Allen NB , et al. Our future: a Lancet commission on adolescent health and wellbeing. Lancet. 2016;387(10036):2423–78. 27174304 10.1016/S0140-6736(16)00579-1 PMC5832967

[19] Jones PB . Adult mental health disorders and their age at onset. Br J Psychiatry. 2013;202(s54):s5–10. 10.1192/bjp.bp.112.119164 23288502

[20] Kessler RC , Amminger GP , Aguilar‐Gaxiola S , Alonso J , Lee S , Ustun TB . Age of onset of mental disorders: a review of recent literature. Curr Opin Psychiatry. 2007;20(4):359–64. 17551351 10.1097/YCO.0b013e32816ebc8c PMC1925038

[21] Das JK , Salam RA , Lassi ZS , Khan MN , Mahmood W , Patel V , et al. Interventions for adolescent mental health: an overview of systematic reviews. J Adolescent Health. 2016;59(4S):S49–60. 10.1016/j.jadohealth.2016.06.020 PMC5026677 27664596

[22] Smith R , Wilkins M . Perinatally acquired HIV infection: Long‐term neuropsychological consequences and challenges ahead. Child Neuropsychol. 2015;21(2):234–68. 24697320 10.1080/09297049.2014.898744

[23] Sohn AH , Hazra R . The changing epidemiology of the global paediatric HIV epidemic: keeping track of perinatally HIV‐infected adolescents. J Int AIDS Soc. 2013;16(1):18555. 23782474 10.7448/IAS.16.1.18555 PMC3687075

[24] Sherr L , Croome N , Parra Castaneda K , Bradshaw K . A systematic review of psychological functioning of children exposed to HIV: using evidence to plan for tomorrow's HIV needs. AIDS Behav. 2014;18(11):2059–74. 24729015 10.1007/s10461-014-0747-6

[25] Skeen S , Sherr L , Tomlinson M , Croome N , Ghandi N , Roberts JK , et al. Interventions to improve psychosocial well‐being for children affected by HIV and AIDS: a systematic review. Vulnerable Child Youth Stud. 2017;12(2):91–116. 29085436 10.1080/17450128.2016.1276656 PMC5659734

[26] Buckley J , Otwombe K , Joyce C , Leshabane G , Hornschuh S , Hlongwane K , et al. Mental health of adolescents in the era of antiretroviral therapy: is there a difference between HIV‐infected and uninfected youth in South Africa? J Adolesc Health. 2020;67(1):76–83. 32269000 10.1016/j.jadohealth.2020.01.010

[27] Kreniske P , Mellins CA , Dolezal C , Korich R , Leu CS , Wiznia A , et al. Sounding the alarm: perinatally HIV‐infected youth more likely to attempt suicide than their uninfected cohort peers. J Adolesc Health. 2019;65(5):702–5. 31481285 10.1016/j.jadohealth.2019.06.006 PMC6814538

[28] Cluver LD , Orkin FM , Campeau L , Toska E , Webb D , Carlqvist A , et al. Improving lives by accelerating progress towards the UN Sustainable Development Goals for adolescents living with HIV: a prospective cohort study. Lancet Child Adolescent Health. 2019;3(4):245–54. 30878118 10.1016/S2352-4642(19)30033-1 PMC6559259

[29] Havens JF , Mellins C . Psychiatric aspects of HIV/AIDS in childhood and adolescence. In: Rutter M , Taylor E , editors. Child and adolescent psychiatry, 5th edn. Oxford, UK: Blackwell; 2008.

[30] Laurenzi CA , Skeen S , Gordon S , Akin‐Olugbade O , Abrahams N , Bradshaw M , et al. Preventing mental health conditions in adolescents living with HIV: an urgent need for evidence. J Int AIDS Soc. 2020;23:e25556. 32869530 10.1002/jia2.25556 PMC7459172

[31] Lowenthal ED , Bakeera‐Kitaka S , Marukutira T , Chapman J , Goldrath K , Ferrand RA . Perinatally acquired HIV infection in adolescents from sub‐Saharan Africa: a review of emerging challenges. Lancet Infect Dis. 2014;14(7):627–39. 24406145 10.1016/S1473-3099(13)70363-3 PMC4074242

[32] Barlow DH . Psychological treatments. Am Psychol. 2004;59(9):869–78. 15584821 10.1037/0003-066X.59.9.869

[33] Craske MG , Anthony MM , Barlow DH . Treatments that work. Mastering your fears and phobias: therapist guide, 2nd edn. Oxford: Oxford University Press; 2006.

[34] Mufson L , Sills R . Interpersonal psychotherapy for depressed adolescents (IPT‐A): an overview. Nordic J Psychiatry. 2006;60(6):431–7. 10.1080/08039480601022397 17162450

[35] Weissman MM , Klerman GL . Interpersonal Psychotherapy for Depression: e‐Book 2015 International Psychotherapy Institute; freepsychotherapybooks.org; 2015. [cited 2020 Oct 10]. Available from: https://www.israpsych.org/wp‐content/uploads/2018/12/interpersonal_psychotherapy_for_depression_‐_myrna_m__weissman_phd.pdf

[36] Hayes SC , Strosahl KD , Wislon KG . Acceptance and commitment therapy: an experiential approach to behavioral change, 2nd edn. New York: The Guilford Press; 2016.

[37] Kieling C , Baker‐Henningham H , Belfer M , Conti G , Ertem I , Omigbodun O , et al. Child and adolescent mental health worldwide: evidence for action. Lancet. 2011;378(9801):1515–25. 22008427 10.1016/S0140-6736(11)60827-1

[38] Betancourt TS , Meyers‐Ohki SE , Charrow A , Hansen N . Annual Research Review: Mental health and resilience in HIV/AIDS‐affected children ‐ a review of the literature and recommendations for future research. J Child Psychol Psychiatry. 2013;54(4):423–44. 22943414 10.1111/j.1469-7610.2012.02613.x PMC3656822

[39] Bhana A , Abas MA , Kelly J , van Pinxteren M , Mudekunye LA , Pantelic M . Mental health interventions for adolescents living with HIV or affected by HIV in low‐ and middle‐income countries: systematic review. BJPsych Open. 2020;6:e104. 32886056 10.1192/bjo.2020.67 PMC7488323

[40] Bucek A , Mellins CA , Leu CS , Dolezal C , Korich R , Wiznia A , et al. Psychiatric disorders and young adult milestones in HIV‐exposed, uninfected youth. AIDS Care. 2020;32(4):420–8. 31537111 10.1080/09540121.2019.1668535 PMC7047564

[41] Moher D , Liberati A , Tetzlaff J , Altman DG . Preferred reporting items for systematic reviews and meta‐analyses: the PRISMA statement. PLoS Medicine. 2009;6:e1000097. 19621072 10.1371/journal.pmed.1000097 PMC2707599

[42] Mayston R , Kinyanda E , Chishinga N , Prince M , Patel V . Mental disorder and the outcome of HIV/AIDS in low‐income and middle‐income countries: a systematic review. AIDS (London, England). 2012;26 Suppl 2:S117–S135. 10.1097/QAD.0b013e32835bde0f 23303434

[43] Ouzzani M , Hammady H , Fedorowicz Z , Elmagarmid A . Rayyan—a web and mobile app for systematic reviews. Syst Rev. 2016;5(1). 10.1186/s13643-016-0384-4 PMC5139140 27919275

[44] Liddle J , Williamson M , Irwig L . Improving health care and outcomes. Method for evaluating research guideline evidence. In: NSW Department of Health S, editor. Australia. 1996.

[45] Dow DE , Mmbaga BT , Gallis JA , Turner EL , Gandhi M , Cunningham CK , et al. A group‐based mental health intervention for young people living with HIV in Tanzania: results of a pilot individually randomized group treatment trial. BMC Public Health. 2020;20(1):3. 32887558 10.1186/s12889-020-09380-3 PMC7487650

[46] Kennard B , Brown L , Hawkins L , Risi A , Radcliffe J , Emslie G , et al. Development and implementation of health and wellness CBT for individuals with depression and HIV. Cognit Behav Pract. 2014;21(2):237–46. 24795524 10.1016/j.cbpra.2013.07.003 PMC4002170

[47] Nestadt DF , Chutima S , McKay MM , Torsak B , Pardo G , Sudrak L , et al. CHAMP+ Thailand: pilot randomized control trial of a family‐based psychosocial intervention for perinatally HIV‐infected early adolescents. AIDS Patient Care STDs. 2019;33(5):227–36. 31067121 10.1089/apc.2019.0021 PMC6531900

[48] Vreeman RC , Nyandiko WM , Marete I , Mwangi A , McAteer CI , Keter A ,, et al. Evaluating a patient‐centred intervention to increase disclosure and promote resilience for children living with HIV in Kenya. AIDS. 2019;33 Supplement 1:S93–101 31397727 10.1097/QAD.0000000000002183

[49] Webb L , Perry‐Parrish C , Ellen J , Sibinga E . Mindfulness instruction for HIV‐infected youth: a randomized controlled trial. AIDS Care. 2018;30(6):688–95. 29067834 10.1080/09540121.2017.1394434 PMC5987527

[50] Willis N , Milanzi A , Mawodzeke M , Dziwa C , Armstrong A , Yekeye I , et al. Effectiveness of community adolescent treatment supporters (CATS) interventions in improving linkage and retention in care, adherence to ART and psychosocial well‐being: a randomised trial among adolescents living with HIV in rural Zimbabwe. BMC Public Health. 2019;19(1):4. 30691425 10.1186/s12889-019-6447-4 PMC6348677

[51] Cavazos‐Rehg P , Byansi W , Xu C , Nabunya P , Sensoy Bahar O , Borodovsky J , et al. The impact of a family‐based economic intervention on the mental health of HIV‐infected adolescents in uganda: results from suubi + adherence. J Adolesc Health. 2021;68(4):742–49. 32980245 10.1016/j.jadohealth.2020.07.022 PMC7987910

[52] Bhana A , Mellins CA , Small L , Nestadt DF , Leu CS , Petersen I , et al. Resilience in perinatal HIV plus adolescents in South Africa. AIDS Care. 2016;28:49–59. 27391999 10.1080/09540121.2016.1176676 PMC4991226

[53] Mon MM , Liabsuetrakul T , Htut KM . Effectiveness of mindfulness intervention on psychological behaviors among adolescents with parental HIV infection: a group‐randomized controlled trial. Asia‐Pacific J Public Health. 2016;28(8):765–75. 10.1177/1010539516675698 27920241

[54] Puffer ES , Green EP , Sikkema KJ , Broverman SA , Ogwang‐Odhiambo RA , Pian J . A church‐based intervention for families to promote mental health and prevent HIV among adolescents in rural kenya: results of a randomized trial. J Consult Clin Psychol. 2016;84(6):511–25. 26985727 10.1037/ccp0000076 PMC4873408

[55] Ssewamala FM , Karimli L , Torsten N , Wang JS , Han CK , Ilic V , et al. Applying a family‐level economic strengthening intervention to improve education and health‐related outcomes of school‐going aids‐orphaned children: lessons from a randomized experiment in Southern Uganda. Prevent Sci. 2016;17(1):134–43. 10.1007/s11121-015-0580-9 PMC4697878 26228480

[56] Thurman TR , Nice J , Luckett B , Visser M . Can family‐centered programing mitigate HIV risk factors among orphaned and vulnerable adolescents? Results from a pilot study in South Africa. AIDS Care. 2018;30(9):1135–43. 29606017 10.1080/09540121.2018.1455957

[57] Li L , Liang LJ , Ji GP , Wu J , Xiao YK . Effect of a family intervention on psychological outcomes of children affected by parental HIV. AIDS Behav. 2014;18(11):2051–8. 24643313 10.1007/s10461-014-0744-9 PMC4169347

[58] Betancourt TS , Ng LC , Kirk CM , Brennan RT , Beardslee WR , Stulac S , et al. Family‐based promotion of mental health in children affected by HIV: a pilot randomized controlled trial. J Child Psychol Psychiatry. 2017;58(8):922–30. 28504307 10.1111/jcpp.12729 PMC5730278

[59] Bell CC , Bhana A , Petersen I , McKay MM , Gibbons R , Bannon W , et al. Building protective factors to offset sexually risky behaviors among black youths: a randomized control trial. J Natl Med Assoc. 2008;100(8):936–44. 18717144 10.1016/s0027-9684(15)31408-5 PMC2536567

[60] McKay MM , Chasse KT , Paikoff R , McKinney LD , Baptiste D , Coleman D , et al. Family‐level impact of the CHAMP family program: a community collaborative effort to support urban families and reduce youth HIV risk exposure. Fam Process. 2004;43(1):79–93. 15359716 10.1111/j.1545-5300.2004.04301007.x

[61] Mellins CA , Nestadt D , Bhana A , Petersen I , Abrams EJ , Alicea S , et al. Adapting evidence‐based interventions to meet the needs of adolescents growing up with HIV in south africa: the vuka case example. Global Soc Welfare. 2014;1(3):97–110. 10.1007/s40609-014-0023-8 PMC4431642 25984440

[62] McKernan McKay M , Alicea S , Elwyn L , McClain ZR , Parker G , Small LA , et al. The development and implementation of theory‐driven programs capable of addressing poverty‐impacted children's health, mental health, and prevention needs: CHAMP and CHAMP+, evidence‐informed, family‐based interventions to address HIV risk and care. J Clin Child Adolescent Psychol. 2014;43(3):428–41. 10.1080/15374416.2014.893519 PMC4215567 24787707

[63] Pardo G , Saisaengjan C , Gopalan P , Ananworanich J , Lakhonpon S , Nestadt DF , et al. Cultural adaptation of an evidence‐informed psychosocial intervention to address the needs of PHIV+ youth in Thailand. Glob Soc Welf. 2017;4(4):209–18. 29104848 10.1007/s40609-017-0100-x PMC5660129

[64] Ssewamala FM , Ismayilova L , McKay M , Sperber E , Bannon W , Alicea S . Gender and the effects of an economic empowerment program on attitudes toward sexual risk‐taking among AIDS‐orphaned adolescent youth in Uganda. J Adolesc Health. 2010;46(4):372–8. 20307827 10.1016/j.jadohealth.2009.08.010 PMC2844862

[65] Ssewamala FM , Sherraden M . Integrating saving into microenterprise programs for the poor: do institutions matter? Soc Serv Rev. 2004;78(3):404–28.

[66] Bhana A , Petersen I , Mason A , Mahintsho Z , Bell C , McKay M . Children and youth at risk: adaptation and pilot study of the CHAMP (Amaqhawe) programme in South Africa. Afr J AIDS Res. 2004;3(1):33–41. 25874981 10.2989/16085900409490316

[67] Petersen I , Mason A , Bhana A , Bell CC , McKay M . Mediating social representations using a cartoon narrative in the context of HIV/AIDS the AmaQhawe family project in South Africa. J Health Psychol. 2006;11(2):197–208. 16464919 10.1177/1359105306061180

[68] Lund C . Poverty and mental health: towards a research agenda for low and middle‐income countries. Comment Soc Sci Med. 2014;111:134–6. 24767968 10.1016/j.socscimed.2014.04.010

[69] Chibanda D , Bowers T , Verhey R , Rusakaniko S , Abas M , Weiss HA , et al. The Friendship Bench programme: a cluster randomised controlled trial of a brief psychological intervention for common mental disorders delivered by lay health workers in Zimbabwe. Int J Mental Health Syst. 2015;9(1):13. 10.1186/s13033-015-0013-y PMC4940904 27408619

[70] Dalgleish T , Black M , Johnston D , Bevan A . Transdiagnostic approaches to mental health problems: current status and future directions. J Consult Clin Psychol. 2020;88(3):179–95. 32068421 10.1037/ccp0000482 PMC7027356

[71] Bruckner TA , Scheffler RM , Shen G , Yoon J , Chisholm D , Morris J , et al. The mental health workforce gap in low‐ and middle‐income countries: a needs‐based approach. Bullet World Health Organ. 2011;89(3):184–94. 10.2471/BLT.10.082784 PMC3044251 21379414

[72] Fairburn CG , Patel V . The impact of digital technology on psychological treatments and their dissemination. Behav Res Ther. 2017;88:19–25. 28110672 10.1016/j.brat.2016.08.012 PMC5214969

[73] Ruzek JI , Yeager CM . Internet and mobile technologies: addressing the mental health of trauma survivors in less resourced communities. Global Mental Health (Cambridge, England). 2017;4:e16. 10.1017/gmh.2017.11 PMC5719483 29230312

[74] Fu Z , Burger H , Arjadi R , Bockting CLH . Effectiveness of digital psychological interventions for mental health problems in low‐income and middle‐income countries: a systematic review and meta‐analysis. Lancet Psychiatry. 2020;7(10):851–64. 32866459 10.1016/S2215-0366(20)30256-X PMC7455253

[75] Myer L . Peer support to mitigate the impact of stigma in young HIV+ pregnant and postpartum women. 2018. Available from: https://www.fic.nih.gov/Grants/Search/Pages/stigma‐hiv‐aids‐r21tw011047.aspx Accessed 13 October 2021

[76] Bachman DeSilva M , Tran NK . Sống vui, Live happily: a psychosocial tele‐health intervention to address multi‐level stigma among youth living with HIV in Vietnam. NICHD; 2019. Available from: https://www.fic.nih.gov/Grants/Search/Pages/stigma‐hiv‐aids‐r21tw011085.aspx

[77] Nalugoda F , Kreniske P , Hofer S , Zhong X , Wei Y , Grilo SA , et al. Cell phones, sexual behaviors and hiv prevalence in Rakai, Uganda: a cross sectional analysis of longitudinal data. AIDS Behav. 2020;24(5):1574–84. 31520238 10.1007/s10461-019-02665-8 PMC7241097

[78] Poushter J , Oates R . Cell phones in Africa: Communication lifeline. Pew Research Center; 2015 [cited 2021 Mar 19]. Available from: https://www.pewresearch.org/global/2015/04/15/cell‐phones‐in‐africa‐communication‐lifeline/

[79] Silver L , Johnson C . Basic Mobile Phones More Common than Smartphones in Sub‐Saharan Africa Pew Research Center. 2019 [cited 2021 Mar 19]. Available from: https://www.pewresearch.org/global/2018/10/09/majorities‐in‐sub‐saharan‐africa‐own‐mobile‐phones‐but‐smartphone‐adoption‐is‐modest/

[80] Kreniske P , Basmajian A , Nakyanjo N , Ddaaki W , Isabirye D , Ssekyewa C , et al. The promise and peril of mobile phones for youth in rural Uganda: multimethod study of implications for health and HIV. J Med Internet Res. 2021;23:e17837. 33528375 10.2196/17837 PMC7886611

[81] Pawson R , Greenhalgh T , Harvey G , Walshe K . Realist review–a new method of systematic review designed for complex policy interventions. J Health Serv Res Policy. 2005;10 Suppl 1:21–34. 16053581 10.1258/1355819054308530

[82] Michie S , Van Stralen MM , West R . The behaviour change wheel: a new method for characterising and designing behaviour change interventions. Implement Sci. 2011;6(1):42. 21513547 10.1186/1748-5908-6-42 PMC3096582

[83] Ramaswamy R , Shidhaye R , Nanda S . Making complex interventions work in low resource settings: developing and applying a design focused implementation approach to deliver mental health through primary care in India. Int J Mental Health Syst. 2018;12(1):7. 10.1186/s13033-018-0181-7 PMC5778695 29387148

[84] Bauer MS , Damschroder L , Hagedorn H , Smith J , Kilbourne AM . An introduction to implementation science for the non‐specialist. BMC Psychol. 2015;3(1):32. 26376626 10.1186/s40359-015-0089-9 PMC4573926

[85] Patel V , Chisholm D , Parikh R , Charlson FJ , Degenhardt L , Dua T , et al. Addressing the burden of mental, neurological, and substance use disorders: key messages from Disease Control Priorities, 3rd edition. Lancet. 2016;387(10028):1672–85. 26454360 10.1016/S0140-6736(15)00390-6

[86] The Lancet Global Health . Mental health matters. Lancet Global Health. 2020;8:e1352. 33069297 10.1016/S2214-109X(20)30432-0 PMC7561290

[87] Lund C , Tomlinson M , De Silva M , Fekadu A , Shidhaye R , Jordans M , et al. PRIME: a programme to reduce the treatment gap for mental disorders in five low‐ and middle‐income countries. PLoS Medicine. 2012;9:e1001359. 23300387 10.1371/journal.pmed.1001359 PMC3531506

[88] Davies T , Lund C . Integrating mental health care into primary care systems in low‐ and middle‐income countries: lessons from PRIME and AFFIRM. Global Mental Health. 2017;4:3. 10.1017/gmh.2017.3 PMC5454718 28596908

[89] Lund C . Improving quality of mental health care in low‐resource settings: lessons from PRIME. World Psychiatry. 2018;17(1):47–8. 29352544 10.1002/wps.20489 PMC5775144

[90] Petersen I , Kemp CG , Rao D , Wagenaar BH , Sherr K , Grant M , et al. Implementation and scale‐up of integrated depression care in South Africa: implementation and scale‐up of integrated depression care in south africa: an observational implementation research protocol. Psychiatric Services. 2021. 10.1176/appi.ps.202000014 PMC8410621 33691487

[91] Accelerate . The UKRI GCRF Accelerating Achievement for Africa's Adolescents Hub UK Research and Innovation. 2021 [cited 2021 Mar 19]. Available from: https://www.acceleratehub.org/

